# Unveiling the impact of allulose on oral microbiota and biofilm formation via a cariogenic potential assessment platform

**DOI:** 10.3389/fcimb.2025.1670139

**Published:** 2025-10-20

**Authors:** Seunghun Han, Kuthirakkal Rajitha, Sungbin Park, Jaeeui Lim, Hee-Young Jung, Junghyun Kim, Dongyeop Kim

**Affiliations:** ^1^ Department of Preventive Dentistry, School of Dentistry, Jeonbuk National University, Jeonju, Republic of Korea; ^2^ Department of Oral Pathology, School of Dentistry, Jeonbuk National University, Jeonju, Republic of Korea; ^3^ Institute of Oral Bioscience, Jeonbuk National University, Jeonju, Republic of Korea

**Keywords:** oral biofilm, extracellular polymeric substances, dental caries, Streptococcus mutans, microcosm

## Abstract

**Introduction:**

The increased consumption of refined carbohydrates, particularly sucrose, has contributed to metabolic disorders and oral diseases such as dental caries by promoting dysbiotic biofilm formation and reducing microbial diversity. Allulose, a rare sugar with physicochemical properties similar to sucrose, has been suggested to offer metabolic health benefits; however, its impact on oral biofilm ecology remains unclear.

**Methods:**

We evaluated the cariogenic potential of allulose using a multi-tiered in vitro platform consisting of single-species planktonic and biofilm models, a dual-species biofilm model involving *Streptococcus mutans* (pathogen) and *Streptococcus oralis* (commensal), and a saliva-derived microcosm biofilm model. Key virulence indicators, including bacterial growth, acid production, biofilm biomass, exopolysaccharide (EPS) synthesis, and microbial community composition, were quantitatively assessed.

**Results:**

Compared to sucrose, glucose, and fructose, allulose supported reduced bacterial growth and acid production, showing a profile similar to non-fermentable sugar alcohols such as xylitol and erythritol. Biofilms developed under allulose conditions lacked the dense EPS-enmeshed microcolonies and dome-shaped architecture characteristic of sucrose-induced S. mutans-dominant biofilms. In the saliva-derived microcosm model, allulose-treated biofilms maintained higher microbial diversity and preserved health-compatible genera such as *Neisseria*, *Haemophilus*, *Veillonella*, and *Granulicatella*.

**Discussion:**

These findings demonstrate that allulose supports lower bacterial virulence activity and minimal biofilm formation compared to common dietary sugars while preserving microbial diversity. This highlights its potential as a non-cariogenic sugar alternative with microbiome-conscious benefits and provides ecological insight into how allulose may modulate oral biofilm structure and function.

## Introduction

The evolution of human diets has been accompanied by an increased refined carbohydrate consumption, which has increased metabolic disorders, such as obesity and diabetes, as well as oral health issues, such as dental caries. The habitual intake of refined sugars, particularly sucrose, promotes oral biofilm formation by reducing microbial diversity and fostering pathogenic microorganisms (Bowen et al., 2018). *Streptococcus mutans* is widely recognized as a primary etiological agent owing to its strong biofilm-forming ability and acidogenic properties (Palmer et al., 2010; Hajishengallis et al., 2017).

Among the fermentable dietary sugars, sucrose plays a crucial role in caries development (Forssten et al., 2010; Sheiham and James, 2015; Benahmed et al., 2021), significantly contributing to dental biofilm accumulation and pathogenicity (Leme et al., 2006; Bowen et al., 2018). It serves as a key substrate for the synthesis of exopolysaccharides (EPS), specifically glucans, catalyzed by glucosyltransferases (Gtfs) secreted by *S. mutans.* These EPS glucans facilitate bacterial adhesion to tooth surfaces, promote biofilm accumulation, and enhance the structural stability of biofilms (Bowen and Koo, 2011; Klein et al., 2015; Kim et al., 2020, 2022). Additionally, water-insoluble glucans trap nutrients and sugars within biofilms, creating an environment conducive to bacterial proliferation.

During sucrose metabolism, *S. mutans* produces acidic metabolites, which reduce the biofilm pH and accelerate enamel demineralization and dental caries progression (Bowen et al., 2018). The metabolic activity of *S. mutans* within dental plaques is central to dental caries development (Parisotto et al., 2010; Durso et al., 2014; Zhang et al., 2022). Although other microorganisms within the biofilm can also be considered cariogenic, *S. mutans* possesses several potential characteristics such as rapid dietary carbohydrate transport and fermentation, acidic byproduct production, extracellular and intracellular polysaccharide synthesis, and stress-responsive carbohydrate metabolism (Banas, 2004; Beighton, 2005; Kanasi et al., 2010).

Considering the critical role of dietary sugars in EPS synthesis and biofilm development, efforts to mitigate dental caries have primarily focused on reducing sugar consumption and enhancing oral hygiene practices (Philip et al., 2018). However, sugar reduction alone is not always feasible owing to dietary habits and lifestyle choices. Consequently, developing non-cariogenic sugar substitutes has attracted attention as an alternative approach. Non-nutritive sweeteners, including synthetic and naturally occurring compounds such as sugar alcohols (polyols), have been widely studied as they interfere with bacterial metabolism and inhibit biofilm formation (Milgrom et al., 2012; Rice et al., 2020; Staszczyk et al., 2020). Despite their potential, the effectiveness of these sugar alternatives varies, causing gastrointestinal side effects and offering limited long-term benefits (Mäkinen, 2016).

D-Allulose, a naturally occurring monosaccharide classified as a rare sugar, is found in small amounts in maple syrup, dried figs or raisins, and brown sugar. It is a fructose epimer via enzymatic treatment with epimerases, with 70% sweetness of sucrose and minimal caloric content (Hu et al., 2021). Clinical studies have suggested that allulose consumption positively influences metabolic health, including improved plasma glucose control, insulin regulation, and weight management, showing benefits in healthy populations and individuals with type 2 diabetes (Hasturk, 2018; Document, 2019; Daniel et al., 2022; Lee et al., 2022). In addition, it has been generally recognized as safe (GRAS) by the United States Food and Drug Administration (FDA), allowing its use as a food ingredient (FDA, 2023).

**Figure 1:**
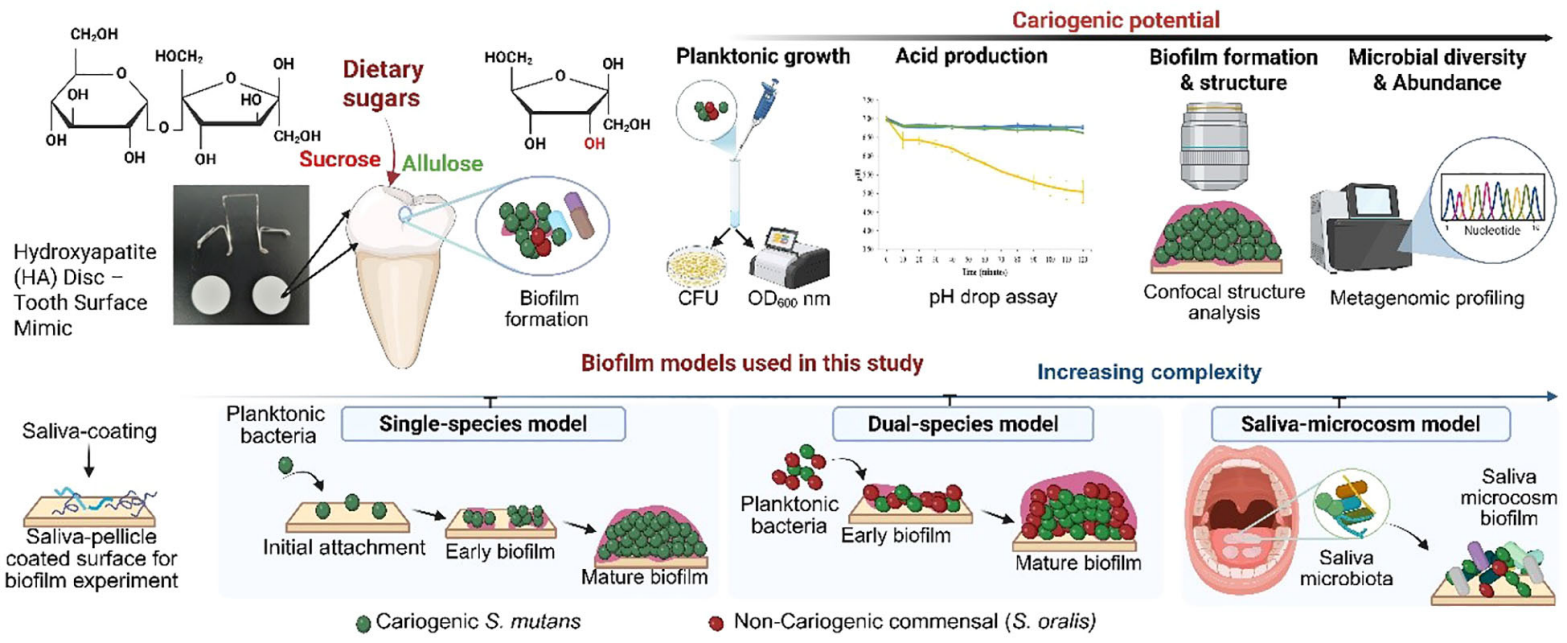
Schematic representation of the overall experimental approach to assess the cariogenic potential of dietary sugars using biofilm models of increasing complexity. The study evaluates the impact of sucrose and allulose on *Streptococcus mutans* biofilm formation and oral microbial communities. The biofilm models progress in complexity: single-species (*S. mutans*), dual-species (*S. mutans* and *Streptococcus oralis*), and a saliva-derived microcosm model that simulates the natural oral environment. This comprehensive approach provides mechanistic insights into the role of dietary sugars in biofilm development and microbial dysbiosis. Figure created using BioRender.

Clinical and controlled feeding studies have shown that allulose is not efficiently metabolized in mammalian systems (Iida et al., 2010; Iwasaki et al., 2018), but its effects on oral microbial ecology—including biofilm interactions—remain poorly explored. Despite the promising attributes of allulose, few studies have explored its effects on oral biofilm formation and microbial diversity. In this study, the effects of dietary carbohydrates on oral microbial cariogenicity were assessed across various ecological models using a multi-tiered platform (**Figure 1**), including single-species planktonic and biofilm, dual-species models involving *S. mutans* and *S. oralis*, and saliva-derived microcosm biofilm experiments with increasing complexity. Each model was used to assess key virulence parameters, including bacterial growth, glycolytic pH drop, biofilm biomass, EPS synthesis, and microbial community composition. The cariogenic potential of allulose has been systematically compared to that of conventional fermentable sugars, such as sucrose, glucose, and fructose (Benahmed et al., 2021), as well as with that of non-fermentable sugar alcohols, such as xylitol and erythritol (Razak et al., 2017; Jeong et al., 2024). Using clinically relevant oral biofilm models, including a hydroxyapatite disc model that mimics the enamel surface and a saliva-based model simulating the oral microbiome, this study provides novel insights into the ecological impact of allulose and highlights its potential as a preventive strategy against dental caries.

## Materials and methods

### Bacterial strains and culture conditions

*S. mutans* UA159 (an established cariogenic dental pathogen and well-characterized EPS producer) was used to generate single- and multi-species biofilms. For inoculum preparation, *S. mutans* was cultured to the mid-exponential phase [optical density at 600 nm (OD_600_) approximately 1.0] in ultrafiltered (10-kDa molecular-mass cutoff membrane; Millipore, MA, USA) tryptone–yeast extract broth [UFTYE; 2.5% tryptone and 1.5% yeast extract (BD Biosciences, San Jose, CA, USA)] with 1% (w/v) glucose at 37°C under 5% CO_2_ as previously described (Kim et al., 2017).

### Sweetener supplementation

The effects of different sweeteners on *S. mutans* biofilm formation and microbial dynamics were evaluated using a panel of common dietary sugars, such as sucrose, glucose, and fructose (Sigma-Aldrich, Saint Louis, MO, USA), and sugar substitutes, such as allulose (Samyang Co., Seongnam, Korea), xylitol (Sigma-Aldrich), and erythritol (Samyang Co.). Each sweetener was freshly prepared at 1% (w/v) final concentration in sterile UFTYE medium based on previous studies demonstrating its physiological relevance in stimulating dietary sugar exposure in the oral cavity (Benahmed et al., 2021). The UFTYE medium (without additional sugars) was used for the baseline comparison, and basal culture medium was used for both planktonic and biofilm models. To replicate normal dietary sugar exposure and oral clearance patterns, the medium was replaced twice daily (at 19 h and 28 h).

### Planktonic growth assay

To assess the cariogenic potential of each sweetener, planktonic growth kinetics and acid production by *S. mutans* were evaluated in UFTYE medium supplemented with 1% glucose, fructose, allulose, xylitol, or erythritol. UFTYE medium without additional sugars served as a blank control. Growth was monitored by measuring the OD_600_ at 30 min intervals for 24 h.

### Glycolytic pH drop assay in planktonic cells

Glycolytic acid production by *S. mutans* was assessed using a pH drop assay as previously described (Jeon et al., 2011; Jung et al., 2022). Briefly, planktonic cultures were harvested, washed once, and resuspended in salt solution (50 mM KCl + 1 mM MgCl_2_). The suspension pH was adjusted to 7.0 using 0.2 M KOH. Each sweetener was added at 1% (w/v) final concentration and the pH was recorded at 10 min intervals over 120 min using a calibrated glass pH electrode (Orion 3-Star, Thermo Scientific, Waltham, MA, USA).

### Gene expression analysis by quantitative real-time polymerase chain reaction

RNA was extracted and purified using protocols optimized for *in vitro* biofilm formation (He et al., 2017). Total RNA was isolated and treated with on-column DNase using a RNeasy Micro kit (Qiagen, Valencia, CA, USA). The RNAs were further treated with a second DNase I (Turbo DNase, Applied Biosystems/Ambion) and purified using the Qiagen RNeasy MinElute Cleanup Kit (Qiagen). Complementary DNA (cDNA) was synthesized from 0.5 µg purified RNA using the iScript cDNA synthesis kit (Bio-Rad Laboratories, Hercules, CA, USA) (Cai et al., 2018). Quantitative real-time polymerase chain reaction (qRT-PCR) was performed using the Applied Biosystems StepOne Real-Time PCR system with gene-specific primers targeting *gtfB*, *gtfC*, *gtfD*, *ftf*, *dexA*, *pdhA*, *adhE*, *ldh*, *atpD*, and *16S rRNA* as previously described (Jeon et al., 2009; He et al., 2017; Cai et al., 2018). Gene expression was analyzed using the comparative ΔΔCt method, normalizing each target gene to 16S rRNA as the internal reference.

### Single-species biofilm model

To replicate the smooth surfaces of the pellicle-coated tooth, biofilms were formed on saliva-coated hydroxyapatite (sHA) disc (surface area: 2.7 ± 0.2 cm^2^) vertically suspended in 24-well plates using a specifically designed wire specimen holder (Xiao et al., 2012; Kim et al., 2018). Filter-sterilized human whole saliva was collected from healthy donors as previously described (Koo et al., 2000). Hydroxyapatite (HA) discs were immersed in cell-free saliva for 1 h to stimulate salivary pellicle formation. The discs were then vertically suspended in a 24-well plate using custom-made holders; inoculated with *S. mutans* (10^5^ colony-forming unit (CFU)/mL; mid-exponential growth phase) in 2.8 mL UFTYE medium supplemented with 1% (w/v) sucrose, glucose, fructose, allulose, xylitol, or erythritol; and incubated at 37°C under 5% CO_2_. The inoculum size reflected the typical *S. mutans* levels in the saliva of caries-active individuals (Ren et al., 2022). The sweetener-containing media were changed at 19 h and 28 h to stimulate eating (meal-like) episodes under continuous sugar exposure. Biofilms were harvested and analyzed at 19, 23, and 43 h post-incubation. Sucrose served as the control (cariogenic reference) for head-to-head comparison.

### Acidogenicity of pre-formed biofilms

To assess the glycolytic activity, a pH drop assay was performed on pre-formed biofilms cultivated on sHA discs. *S. mutans* biofilms were grown for 43 h in UFTYE medium supplemented with 1% (w/v) sucrose. At 43 h, the discs were transferred to fresh solutions containing 1% (w/v) sweetener, and pH was recorded at 10-min intervals over 120 min to monitor acid production. The initial rate of acid production, which is considered the best indicator of the acid production capacity of the biofilm, was determined from the pH values.

### Dual-species biofilm model

A dual-species biofilm model was developed using the cariogenic pathogen *S. mutans* UA159 and oral commensal *S. oralis* KCTC 13038 [originated from ATCC 35037; obtained from Korean Collection for Type Cultures (KCTC), Jeongeup, Korea]. Bacterial suspensions were prepared and mixed to obtain final inoculum concentrations of 10^5^ and 10^7^ CFU/mL for *S. mutans* and *S. oralis*, respectively. Consistent with the ecological plaque hypothesis, this mixed inoculum was cultured in UFTYE medium containing 0.1% (w/v) sucrose (cariogenic reference) for 19 h to establish an initial colonization community. The discs were then transferred to UFTYE containing 1% sucrose to stimulate a cariogenic challenge at 19 h. The culture medium was changed at 28 h, and the biofilms were harvested at 43 h to determine the viable bacterial count, expressed as CFU per biofilm, using blood agar plating.

### Saliva-derived microcosm biofilm model

To simulate a clinically relevant oral microenvironment, a saliva-derived microcosm biofilm model was established using sHA discs (Liu et al., 2023) with slight modifications. The saliva-originated microbial consortium was centrifuged at 3,000× *g* for 10 min to remove the host cells, and the salivary microbiome (saliva collected from healthy individuals, as qualified *S. mutans* absence) was inoculated for initial binding (1 h). Next, approximately 10^5^ CFU/mL *S. mutans* was inoculated into UFTYE medium containing 1% (w/v) sucrose or allulose, or UFTYE medium without additional sugars (blank).

### Biofilm imaging using confocal microscopy

The biofilms formed under each condition were examined using confocal microscopy. The bacterial cells were stained with 2.5 μM SYTO 9 green-fluorescent nucleic acid stain (485/498 nm; Molecular Probes Inc., Eugene, OR, USA), while EPS was labeled with 1 μM Alexa Fluor 647–dextran conjugate (647/668 nm; Molecular Probes Inc.) The 3D biofilm architecture was acquired using a C2+ confocal microscope (Nikon, Tokyo, Japan) with 20× (0.75 numerical aperture (NA)). NIS-Elements software version 5.21 (Nikon) was used to create 3D renderings to visualize the biofilm architecture (Jung et al., 2022).

### Metagenome profiling of saliva-derived microcosm biofilms

Microcosm biofilm samples were collected from the sHA discs and eluted in phosphate-buffered saline (PBS). Genomic DNA was extracted using the FastDNA^®^ Spin Kit for Soil (MP Biomedicals, USA) and quantified using a BioTek Epoch™ spectrophotometer. DNA quality was verified using 1% agarose gel electrophoresis. The V3–V4 region of the bacterial *16S rRNA* gene was amplified using the universal primers 341F and 805R with overhang Illumina adapter sequences following the Nextera™ consensus design. Polymerase chain reaction (PCR) amplification was conducted in two steps: the first round of target amplification and the second round of indexing. The first PCR included 25 cycles using Takara Ex Taq polymerase, and the second PCR consisted of eight indexing cycles. Libraries were purified using AMPure XP beads (Beckman Coulter) and quantified using the Quant-iT PicoGreen dsDNA Assay Kit. Library quality was assessed using an Agilent 2100 Bioanalyzer, and sequencing was performed using the Illumina MiSeq platform with the MiSeq Reagent Kit v2 (500 cycles). Chimeric sequences were detected and removed using the UCHIME method and embedded in the EzBioCloud database (Yoon et al., 2017). Downstream analysis included alpha- and beta-diversity metrics (e.g., Shannon index and Bray–Curtis distance) and relative abundance profiling across taxonomic ranks. The *16S rRNA* gene sequences are available in the NCBI Sequence Read Archive (BioProject accession number: PRJNA1269248).

### Statistical analysis

All data are presented as mean ± standard deviation (SD). For comparisons involving multiple groups against a single control, a one-way analysis of variance (ANOVA) followed by Dunnett’s multiple comparison test was applied. Interspecies CFU comparisons were analyzed using non-parametric Kruskal–Wallis tests with Dunn’s *post-hoc* correction. For gene expression analysis, ΔCt values were evaluated using one-way ANOVA followed by Holm–Šídák’s multiple comparisons test. Statistical significance was set at *p*<0.05. Analyses were performed using GraphPad Prism version 10.4.0 (GraphPad Software, San Diego, CA, USA).

## Results and discussion

### Planktonic growth and acid production of *S. mutans* in response to various sweeteners

**Figure 2:**
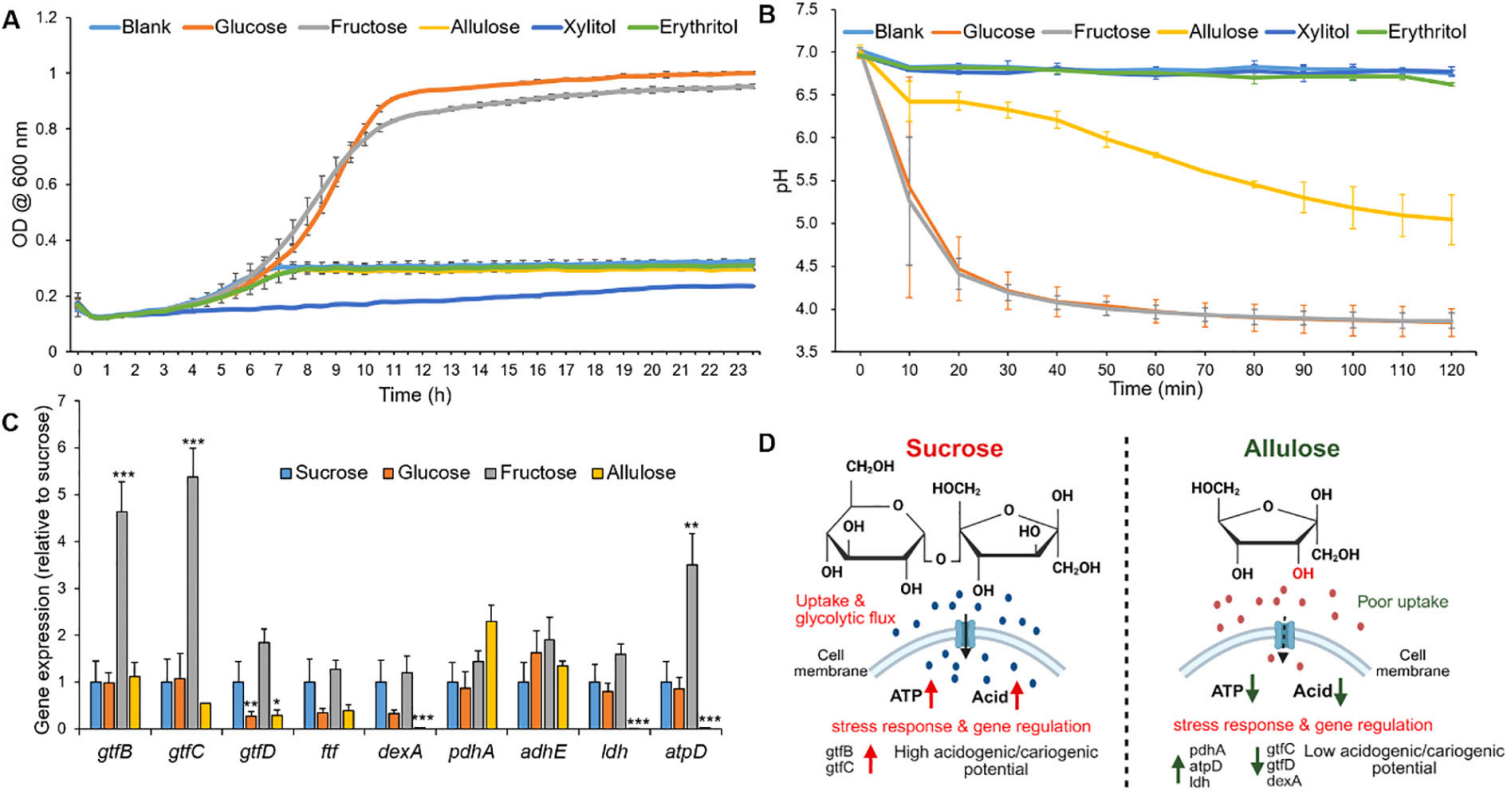
Bacterial growth curve, glycolytic pH drop, and relative gene expression of *Streptococcus mutans* in response to different sweeteners. **(A)** Growth curve of *S. mutans* measured as optical density at 600 nm (OD_600_) over time in a UFTYE medium supplemented with 1% (w/v) glucose, fructose, allulose, xylitol, or erythritol. The UFTYE medium without any sugar supplementation was used as a blank control. **(B)** pH drop assay of *S. mutans* in the presence of different sweeteners. Different sweeteners were added to 50 mM KCl + 1 mM MgCl_2_ solution (pH=7) to obtain a concentration of 1% (w/v), and pH changes were assessed over 120 min. The data are presented as mean ± standard deviation (n=3). **(C)** Relative gene expression of *S. mutans* planktonic cells in response to different sweeteners at 19 h. Bar graph shows the relative expression levels of key genes associated with biofilm formation (*gtfB*, *gtfC*, *gtfD*, *ftf*), extracellular matrix (*dexA*), energy metabolism (*pdhA*, *adhE*, *atpD*), and acid production (*ldh*) in *S. mutans* cultured in UFTYE medium supplemented with 1% (w/v) sucrose, glucose, fructose, or allulose. Xylitol and erythritol were excluded from gene expression analysis due to minimal growth, which precluded reliable RNA extraction. Data represent mean ± standard deviation from biological replicates (n=6). Only statistically significant differences compared to the sucrose group are indicated with asterisks: *p<0.05; **p<0.01; ***p<0.001. All other comparisons were not significant (ns). **(D)** Conceptual working model summarizing observed phenotypes under sucrose versus allulose, including bacterial growth, glycolytic pH drop, EPS production, and gene expression profiles. The schematic is illustrative and created using BioRender.

To evaluate allulose with fermentable sugars and non-fermentable polyols in a controlled setting, the initial phase of the cariogenic evaluation platform concentrated on a single-species model using the key cariogenic pathogen *S. mutans* (Hajishengallis et al., 2017; Kim et al., 2020). To assess how different sweeteners affect the planktonic growth kinetics (OD_600_) and the acid production (pH drop assay), *S. mutans* was cultured in a UFTYE defined medium supplemented with 1% (w/v) sweetener. Basal medium UFTYE (blank) is a complex medium that contains low-molecular-weight nutrients (<10 kDa). The minimal growth observed in the UFTYE medium without additional sugars likely reflects the utilization of these residual nutrients. The growth curves showed that glucose and fructose supported robust bacterial growth with an extended exponential phase, reaching the stationary phase (OD_600_: approximately 1.0, **Figure 2A**). These observations align with previous findings that fermentable carbohydrates serve as a preferred and rapidly metabolizable energy source that fuels bacterial proliferation (Jurakova et al., 2023). Moreover, these sugars induced a steep pH drop, with the final pH value dropping to 4.20 ± 0.04 within 30 min, indicating high acid production from glycolytic fermentation (**Figure 2B**).

Conversely, although allulose had a structural resemblance to fructose, it did not promote exponential growth and exhibited only marginal acidification (**Figures 2A, B**). The growth in the presence of allulose supplementation remained constant at OD_600_ approximately 0.3, and the pH remained above 5.0 (critical pH for enamel demineralization) over 120 min with a reduction in acid production by 99% compared to that in the presence of sucrose, indicating the absence of key metabolic pathways for effectively metabolizing allulose. This growth profile highlights that, under the conditions tested, allulose supported minimal bacterial growth and acid production compared to conventional sugars. Xylitol and erythritol also failed to support exponential growth and acid production, establishing their roles as non-fermentable sugar alcohols (**Figures 2A, B**) (Mäkinen, 2010; Jeong et al., 2024).

### Dynamics of cariogenicity-associated genes in response to allulose and other fermentable sugars

To elucidate the molecular basis underlying the observed differences in planktonic growth and acid production, we examined the expression of key virulence genes in 19 h-old *S. mutans* cells cultured in the presence of different dietary sweeteners (**Figure 2C**). Planktonic gene expression is important because it may indicate the ability of free cells to colonize a pre-formed biofilm or a new surface (Durso et al., 2014). The cariogenicity of *S. mutans* is closely linked to its ability to synthesize extracellular glucans and produce acids via carbohydrate fermentation.

Glucosyltransferase (Gtf catalyzes EPS synthesis and forms a protective scaffold that supports biofilm integrity under external stress (Wang et al., 2018). Specifically, *gtfB* and *gtfC* are associated with insoluble and soluble glucan production, whereas *gtfD* contributes to soluble glucan production (Zhao et al., 2014). In our study, *gtfB* and *gtfC* expression were significantly upregulated in the presence of fructose than in the presence of sucrose (*p*<0.0001), consistent with prior findings on fructose-mediated EPS-related gene induction (Shemesh et al., 2006). In contrast, *gtfD* was significantly downregulated in the presence of allulose (*p*<0.05), which may limit the primer availability for initial EPS synthesis, thereby impairing biofilm formation.

Notably, *gtfB* expression remained unaffected by allulose exposure. Since environmental stress often triggers *gtfB* upregulation to promote adhesion and glucan synthesis (Zhang et al., 2022), its stable expression in the presence of allulose may represent a compensatory mechanism for surface attachment in nutrient-limited or metabolically inactive states. It is important to note that transcriptional levels may not always correspond to enzymatic activity because post-transcriptional regulation can modulate the final protein function (Zhang et al., 2021).

Unlike *gtf* genes, *ftf* expression, which is involved in fructan synthesis, remained unchanged in the presence of all sweeteners, suggesting a limited role of fructan-mediated EPS under these experimental conditions. Interestingly, the significant downregulation of *dexA*, which encodes the dextranase responsible for glucan degradation during biofilm remodeling, suggests that, under allulose conditions, EPS synthesis and biofilm maturation pathways were less active compared to fermentable sugars (Igarashi et al., 2002).

*pdhA*, which encodes a component of the pyruvate dehydrogenase complex, was upregulated in the presence of allulose. This enzyme links glycolysis to the tricarboxylic acid (TCA) cycle by converting pyruvate into acetyl-CoA, suggesting a shift toward alternative energy metabolism in response to inefficient allulose fermentation. This metabolic reprogramming was consistent with previous findings under carbohydrate-limited conditions (Lemos and Burne, 2008), indicating that under allulose conditions, *S. mutans* exhibited a low-metabolic, low-virulence-like state.

In terms of acidogenicity, the expression of *ldh*, which encodes lactate dehydrogenase, a key enzyme in lactic acid production, was significantly lower in the presence of allulose than in the presence of sucrose (*p*<0.001), correlating with the reduced acid production. Similarly, *atpD*, encoding the β-subunit of the F_1_F_0_–ATPase complex responsible for proton extrusion under acidic conditions, was highly expressed in the presence of fructose (*p*<0.01), reflecting increased acid stress. Conversely, its significant downregulation in the presence of allulose (*p*<0.001) supports the notion that allulose imposes minimal acidogenic stress, further reinforcing its low cariogenic potential.

Taken together, the gene expression profiling and phenotypic data on growth and acid production support a less-cariogenic profile for allulose under the tested conditions (**Figure 2D**). Its inability to activate key virulence pathways, including those involved in EPS synthesis (*gtfD*), acid production (*ldh*), and acid tolerance (*atpD*), aligns it with the properties of non-cariogenic sugar alcohols, such as xylitol and erythritol (Mäkinen, 2011; Milgrom et al., 2012; Runnel et al., 2013; De Cock, 2018).

### Modulation of *S. mutans* biofilm formation and EPS synthesis by sweeteners

**Figure 3:**
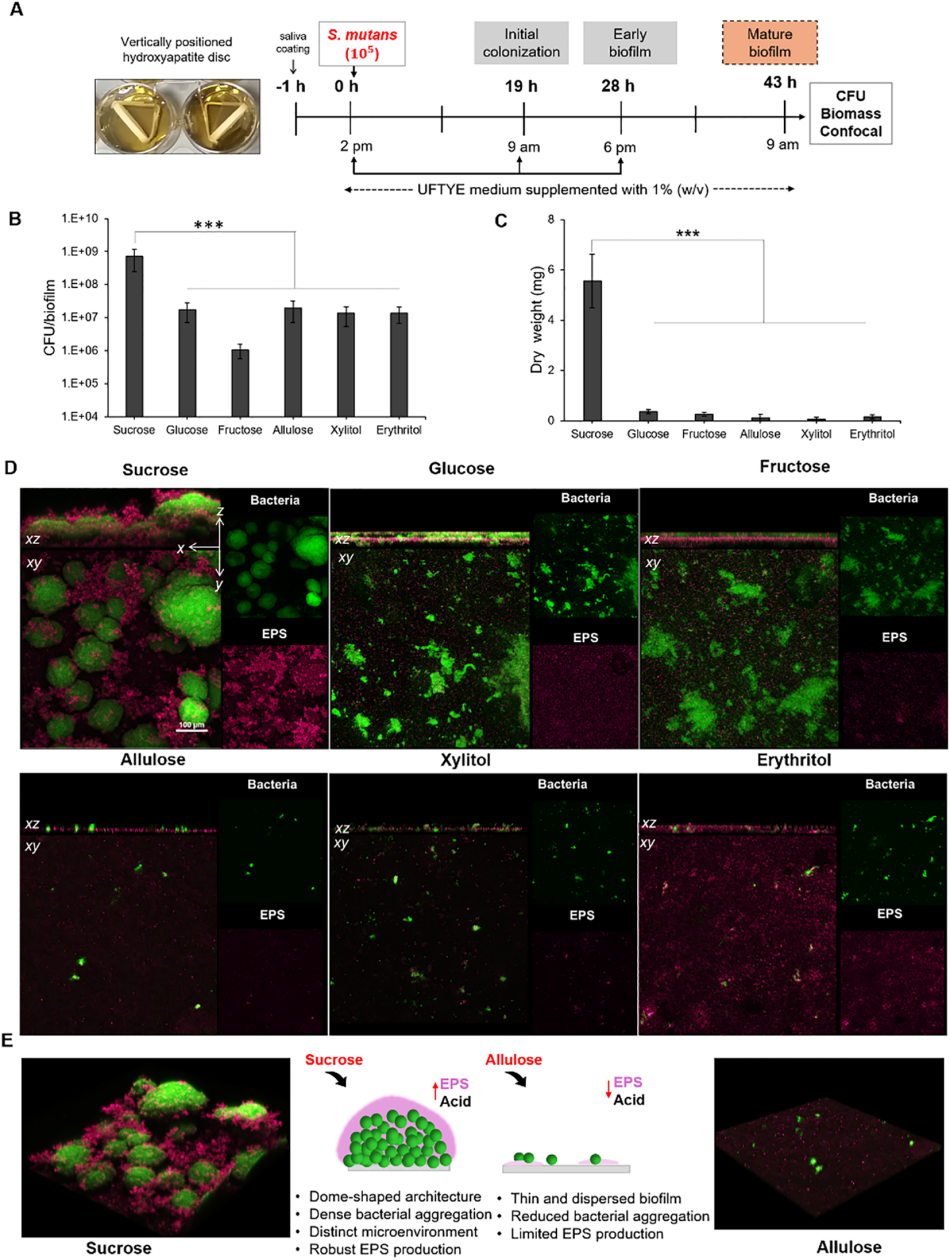
Experimental workflow and microbiological analysis of *Streptococcus mutans* biofilm architecture at 43 h. **(A)** Experimental design for *S. mutans* biofilm formation on the saliva-coated hydroxyapatite (sHA) discs. Biofilms were cultured in UFTYE media supplemented with 1% (w/v) sweetener at 37°C and 5% CO_2_. The culture medium was changed twice daily (at 19 h and 28 h). Biofilm quantification in terms of **(B)** colony-forming units (CFU)/biofilm and **(C)** dry weight. **(D)** Confocal images showing the structural and spatial organization of 43-h-old *S. mutans* biofilm grown in the presence of various sweeteners. **(E)** Representative 3D confocal microscopy images showing biofilm formation in the presence of sucrose (left) and allulose (right). Green represents bacterial cells, while magenta indicates the extracellular polysaccharide (EPS) matrix. The data are presented as mean ± standard deviation (n=5). One-way analysis of variance (ANOVA) followed by Dunnett’s multiple comparisons test was used to compare each treatment group with the sucrose control. ***p<0.0001.

To assess the effects of dietary sugars on biofilm development and EPS production, which are key indicators of cariogenic potential, we employed a sHA disc model to simulate a pellicle-formed tooth surface (**Figure 3A**). *S. mutans* was incubated for 43 h in the presence of 1% (w/v) various sweeteners, and the mature biofilm biomass was quantified in terms of CFU and dry weight (**Figures 3B, C**). The three-dimensional (3D) architecture of the biofilm, consisting of both bacterial cells and EPS, was visualized using confocal microscopy (**Figure 3D**).

Quantitative data (**Figures 3A, B**) showed that sucrose yielded significantly higher CFU counts and dry biofilm weights (*p*<0.001) than all the other sugars. This further validates its established role as a high-carbohydrate sugar that enhances bacterial cell attachment and EPS production (Banas and Vickerman, 2003; Duarte et al., 2008; Razak et al., 2017). The dense, well-defined, dome-shaped microcolonies embedded in the EPS matrix under sucrose supplementation (**Figures 3D, E**) were consistent with those reported in previous reports on the sucrose-driven upregulation of Gtf exoenzymes, which are crucial for water-insoluble glucan synthesis and robust biofilm development. In contrast, supplementation with other fermentable sugars, such as glucose and fructose, resulted in comparatively less biomass with a dispersed cell–EPS arrangement (**Figure 3D**). These results indicate that, although these monosaccharides are fermentable, they cannot replace the dual role of sucrose as a fuel for acid production and a key substrate for water-insoluble EPS biosynthesis.

Biofilms developed in the presence of allulose showed significantly reduced CFU counts and dry weights, similar to those developed in the presence of xylitol and erythritol (**Figures 3A, B**). Confocal imaging further showed that biofilms formed under allulose conditions lacked the dense, matrix-rich structures typical of sucrose-induced *S. mutans* biofilms (**Figures 3D, E**). The initial inhibition of biofilm formation in the presence of allulose can be attributed to reduced planktonic growth compared to that in the presence of fermentable sugars, correlating with the gene expression data showing a more complicated adaptive response. The upregulation of genes associated with metabolic stress and compensatory energy pathways (*pdhA* and *adhE*, respectively; **Figure 2C**) indicates that *S. mutans* may activate adaptive strategies under nutrient-limited conditions rather than simply undergoing growth arrest. These results suggest that biofilms under allulose conditions were characterized by reduced biomass, less EPS, and altered metabolic programming compared to fermentable sugars.

Taken together, the results of the single-species *S. mutans* biofilm provide consistent evidence that, under the conditions tested, allulose exhibits fewer cariogenic phenotypes compared to fermentable sugars and shows functional similarities to previously reported sugar alcohols (Miyasawa‐Hori et al., 2006; Park et al., 2014). Our study further showed that biofilm architecture and EPS accumulation are the main indicators of cariogenic potential, and the sHA biofilm model is pertinent for evaluating dietary sugar under near-physiological conditions.

### Acidogenic potential of sweeteners in mature *S. mutans* biofilm

**Figure 4:**
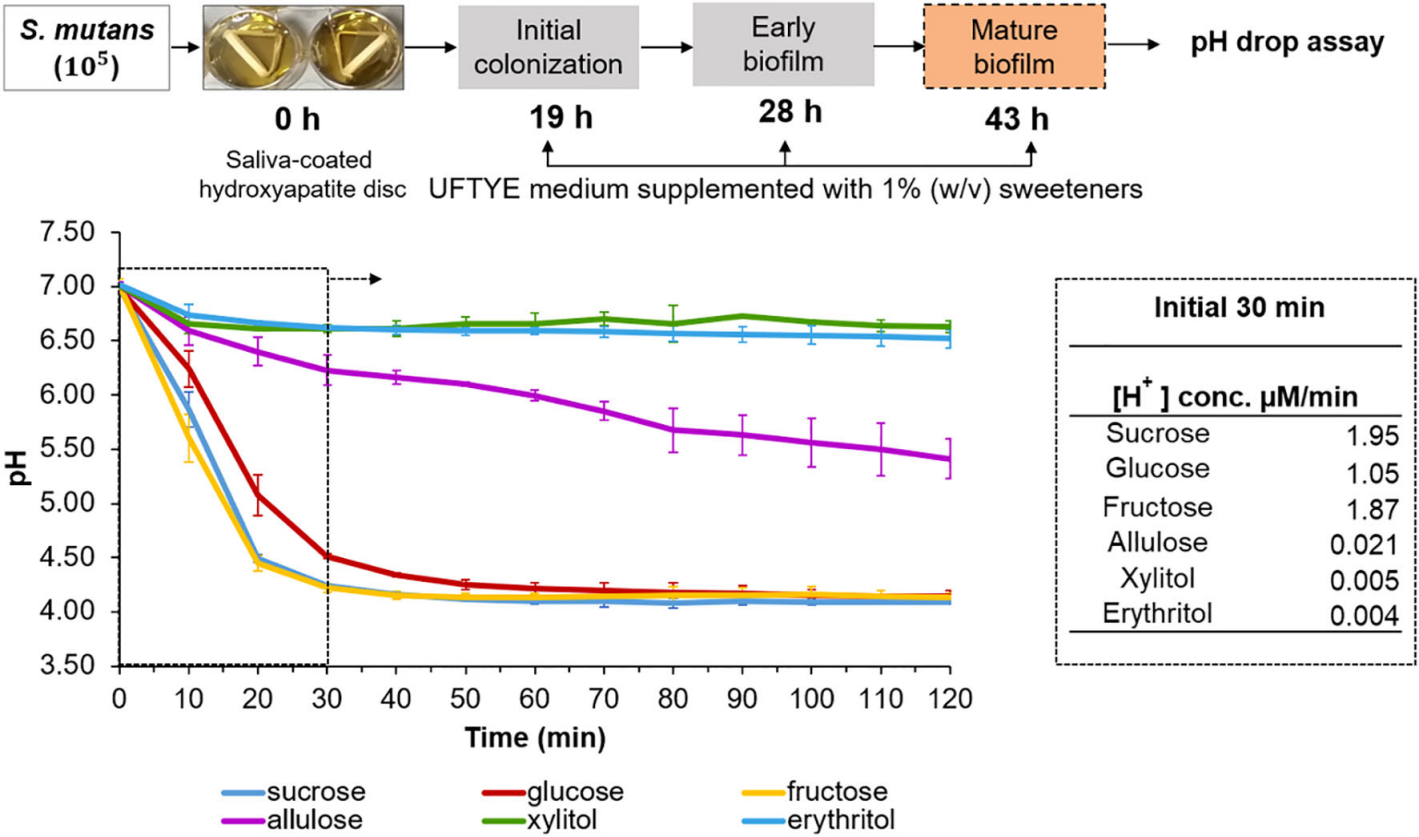
pH drop assay with pre-formed *Streptococcus mutans* biofilm in the presence of different sweeteners. The top panel illustrates the experimental design where *S. mutans* biofilms were grown on saliva-coated hydroxyapatite (sHA) discs in UFTYE medium supplemented with 1% (w/v) sucrose. The pH drop assay was performed at 43 h in the presence of different sweeteners. The bottom panel presents the pH drop over time, showing acid production from bacterial metabolism. Data are presented as the mean of two biologically different experiments.

Assessing how pre-formed oral biofilms respond to altered sugar exposure is necessary to comprehend how dietary sugar variations affect the cariogenic behavior of established biofilms. This was evaluated by a glycolytic pH drop assay using a 43 h-old *S. mutans* biofilm grown on tooth mimetics (**Figure 4**). To mimic the dietary changes, biofilms grown in the presence of 1% sucrose were exposed to various fermentable and non-fermentable sweeteners, and the pH drop was monitored for 120 min. The results show that sucrose supports the highest H^+^ ion release (1.95 µM/min) and reaches a pH of 4.5 within 20 min of exposure, which further establishes its role as a fuel for both bacterial metabolisms, followed by acid production and EPS-associated proton accumulation (Duarte et al., 2008; Razak et al., 2017; Jurakova et al., 2023). The acidogenic profile of fructose was similar to that of sucrose than that of glucose, which is consistent with earlier findings on the expression of fructose-induced virulence factors in *S. mutans* (Shemesh et al., 2006).

Conversely, allulose-, xylitol-, or erythritol-supplemented biofilms maintained near-neutral pH throughout the assay period, with 98%, 99%, and 99% reduction in acid production, respectively, within 30 min compared to the sucrose-supplemented biofilm (**Figure 4**). This result confirms that they serve as poorly fermentable substrates for *S. mutans* metabolism.

Although the oral cavity generally maintains a near-neutral pH, localized acidification from dietary sugars can promote enamel demineralization, favor acid-tolerant microbiota proliferation, and disrupt biofilm homeostasis (Marsh, 1994; Welin-Neilands and Svensater, 2007; Ikäläinen et al., 2024). Poor oral hygiene may exacerbate these effects by allowing the aciduric biofilms to mature and persist (Sälzer et al., 2020). In this context, alternative sweeteners, such as allulose, may help suppress acid accumulation, even in 3D-structured biofilms.

### Modulation of interspecies balance in a dual-species biofilm model by sweeteners

**Figure 5:**
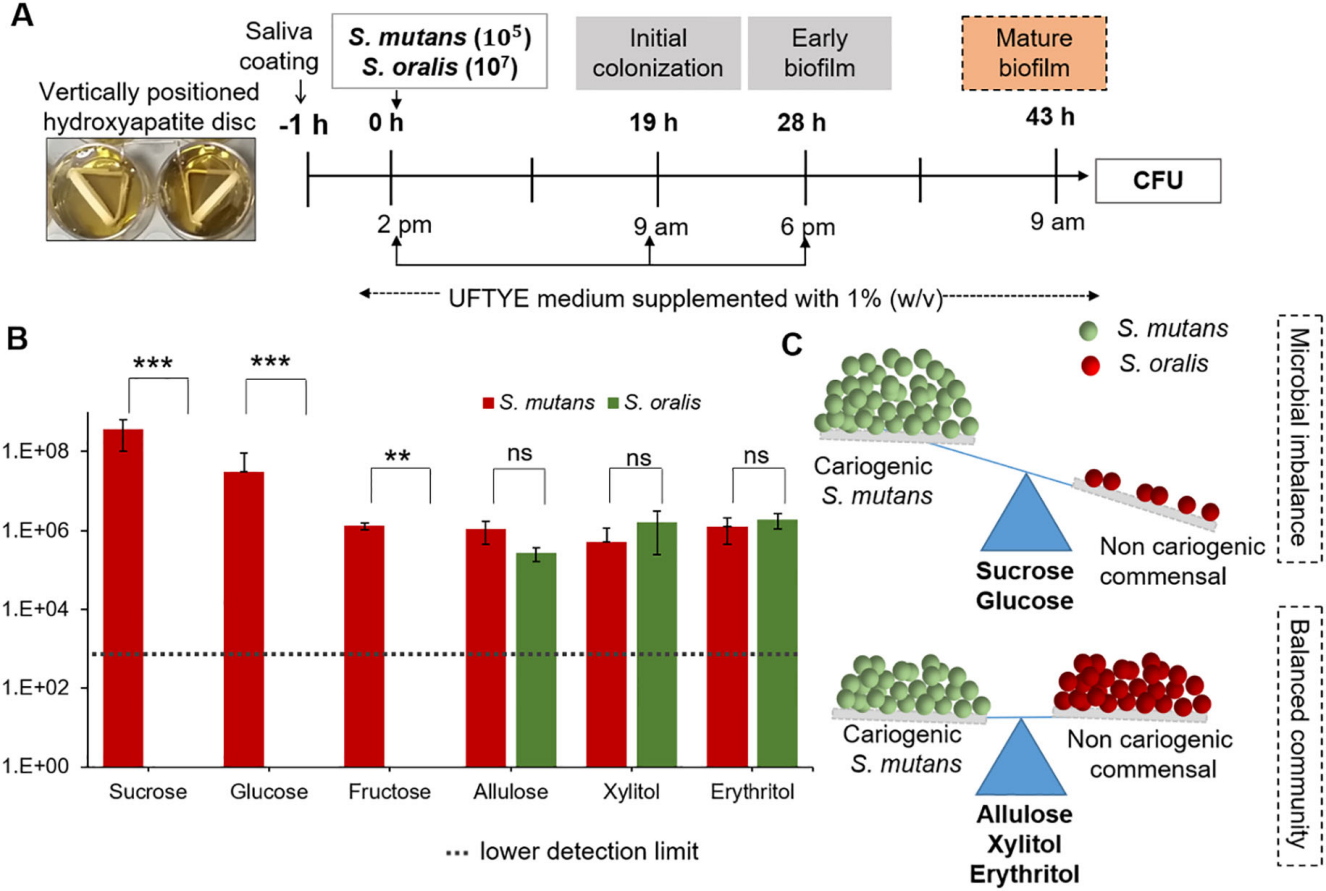
Impact of different sweeteners on *Streptococcus mutans* and *Streptococcus oralis* interspecies biofilm formation. **(A)** Experimental design for interspecies biofilm formation of *S. mutans and S. oralis* saliva-coated hydroxyapatite (sHA) discs. The biofilms were cultured in UFTYE medium supplemented with 1% (w/v) sweetener at 37°C and 5% CO_2_. **(B)** Quantification of *S. mutans* and *S. oralis* colony-forming units (CFU) in 43 h-old biofilms grown in the presence of different sweeteners. **(C)** Schematic representation of microbial balance shift induced by different sweeteners, illustrating the impact of sugar supplementation on the dominance of *S. mutans* (cariogenic) and *S. oralis* (commensal species). Data are presented as mean ± standard deviation (n=8). Pairwise comparisons between *S. mutans* and *S. oralis* in the presence of each sweetener were performed using Dunn’s multiple comparisons test following Kruskal–Wallis analysis. ***p<0.0001; **p<0.001; ns, not significant.

Microbial interactions play crucial roles in the modulation of health and disease. Cooperative and competitive interactions among oral pathogens and commensals, as well as their composition, are modulated by ecological factors, such as nutrients and pH (Marsh, 2003). In the second phase of our cariogenic assessment platform, we evaluated the impact of dietary sugar (as it is the major nutrient source) on the dual-species biofilm model using *S. mutans* as the oral pathogen and *S. oralis* as the commensal bacterium (**Figure 5A**). The results show that supplementation with 1% (w/v) sucrose, glucose, or fructose selectively enriched *S. mutans* over *S. oralis* (*p*<0.001, **Figure 5B**). *S. mutans* enrichment in the presence of fermentable sugars such as sucrose, glucose, and fructose is driven by its robust metabolic and ecological adaptations. *S. mutans* can produce acid and survive in low-acid conditions because of its highly efficient fermentable sugar uptake system and virulence-associated gene expression. Particularly, sucrose can serve as both a substrate and precursor for EPS matrix synthesis for dense, acidic, and robust biofilm formation (Koo et al., 2010). Previous reports have shown that *S. mutans* can compete with commensal species, such as *S. oralis*, and dominate biofilms through bacteriocin production and a quorum-sensing mechanism (Li et al., 2002; Kreth et al., 2005). Conversely, *S. oralis* is an early colonizer associated with healthy oral biofilms, sensitive to acidic conditions, and lacks EPS production capacity, which makes it vulnerable to sugar-rich and acidic environments. This clearly shows the ecological imbalance that can shift the biofilm into a dysbiotic state with enhanced cariogenic potential.

In contrast, allulose, xylitol, and erythritol maintained a balanced microbial composition, with no significant overgrowth of *S. mutans* toward *S. oralis* (**Figure 5B**). Previous studies have shown that the balance of *S. oralis* abundance in oral biofilms can be beneficial for dental health because these commensals secrete hydrogen peroxide, which can interfere with *S. mutans* growth and colonization (Kim et al., 2022). These findings align with the ecological plaque hypothesis, indicating the importance of non- or less-fermentable sugars such as allulose in reducing acid production and suppressing the overgrowth of acid-tolerant species over that of sensitive commensal bacteria in oral biofilms (Marsh, 1994, 2003). Therefore, oral microbial homeostasis and cariogenic biofilm formation control depend on the preservation of the balance between *S. mutans* and *S. oralis* (**Figure 5C**).

### Ecological modulation in saliva-derived multi-species microcosm biofilm model by allulose

**Figure 6:**
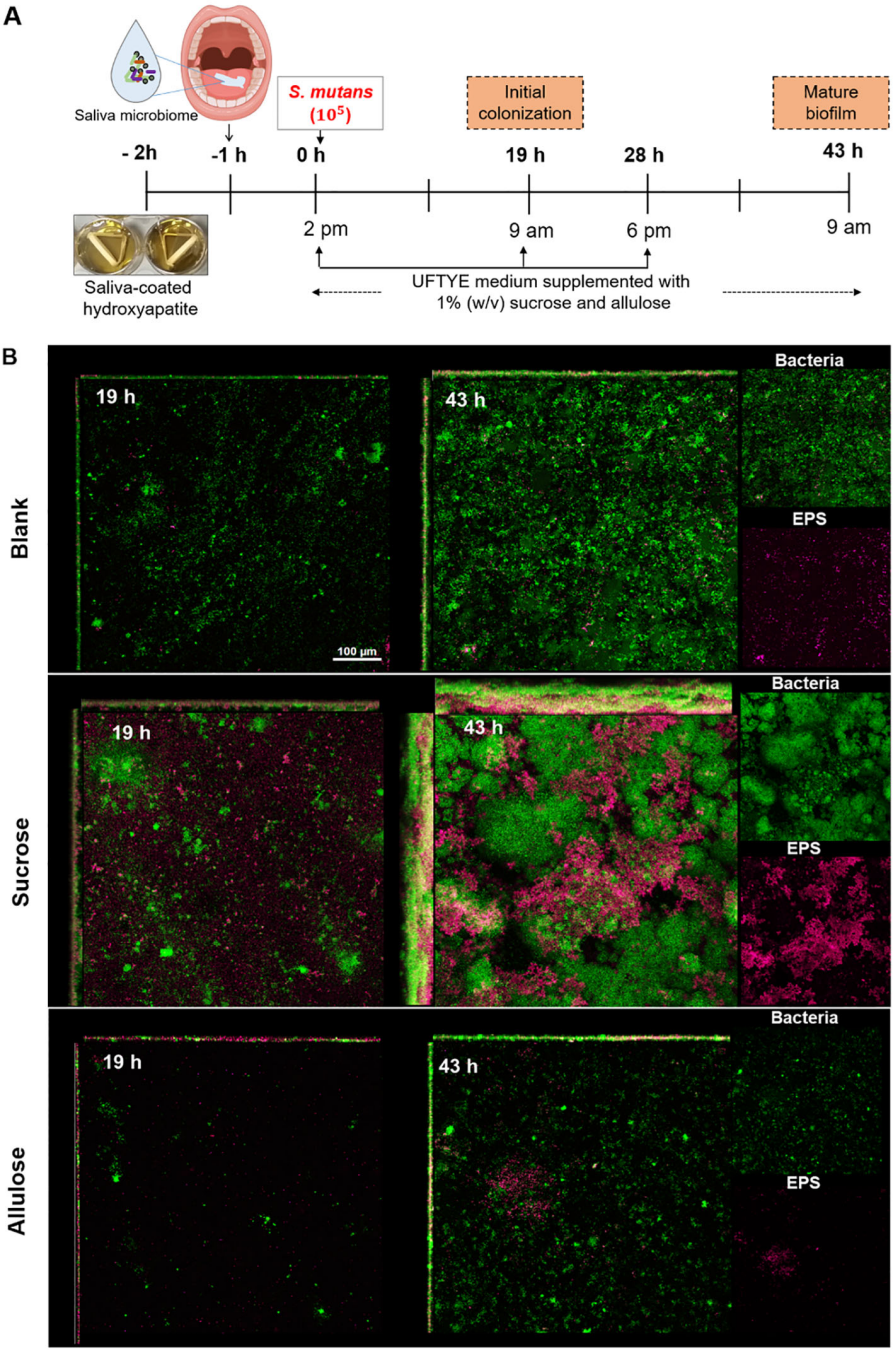
Biofilm development and extracellular polysaccharide (EPS) production in a saliva-derived microcosm model in the presence of 1% (w/v) sucrose or allulose. **(A)** Schematic representation of an ex vivo microcosm biofilm model using saliva-coated hydroxyapatite (sHA) discs. The discs were first incubated with the saliva from healthy donors for 1 h to allow initial microbial colonization, followed by incubation in UFTYE media supplemented with 1% (w/v) sucrose or allulose and inoculated with *S. mutans*. UFTYE media without any sweetener supplementation served as the blank control. **(B)** Confocal laser scanning microscopy images of biofilms grown at 19 and 43 h. Green and magenta represent bacterial cells and the extracellular polysaccharides (EPS), respectively.

Next, we used HA discs coated with pooled whole saliva to stimulate a clinically relevant polymicrobial environment and support early colonization by native microbes. The ecological response to allulose was evaluated under caries-promoting conditions by supplementation with 1% (w/v) sucrose, followed by adding *S. mutans*, and compared with that of the no-added-carbohydrate saliva control. Changes in biofilm structure and community profile were examined in early (19 h) and mature (43 h) biofilms (**Figure 6A**).

Confocal imaging revealed significant architectural differences between the biofilms grown under sucrose and allulose supplementation. Early biofilm maturation, in terms of significant bacterial adhesion and EPS deposition, was observed in sucrose-supplemented biofilms at 19 h (**Figure 6B**). In contrast, the saliva-only control (without sugar addition) showed moderate bacterial attachment with little or no EPS, whereas the allulose-supplemented biofilms showed sparse bacterial colonization with minimal EPS deposition. Minimal baseline growth or biofilm may have resulted from trace amounts of sugars in the UFTYE medium; however, all groups were cultured under the same basal medium to ensure comparability. Upon extended incubation for 43 h, sucrose induced the development of dense dome-shaped microcolonies with a robust EPS matrix. The allulose-supplemented biofilm maintained a reduced biofilm structure with minimal bacteria and EPS, suggesting that biofilms under allulose conditions remained thinner and less structured over time compared to sucrose-treated biofilms; as expected, biofilms developed in the saliva-only group remained less organized and thinner.

**Figure 7:**
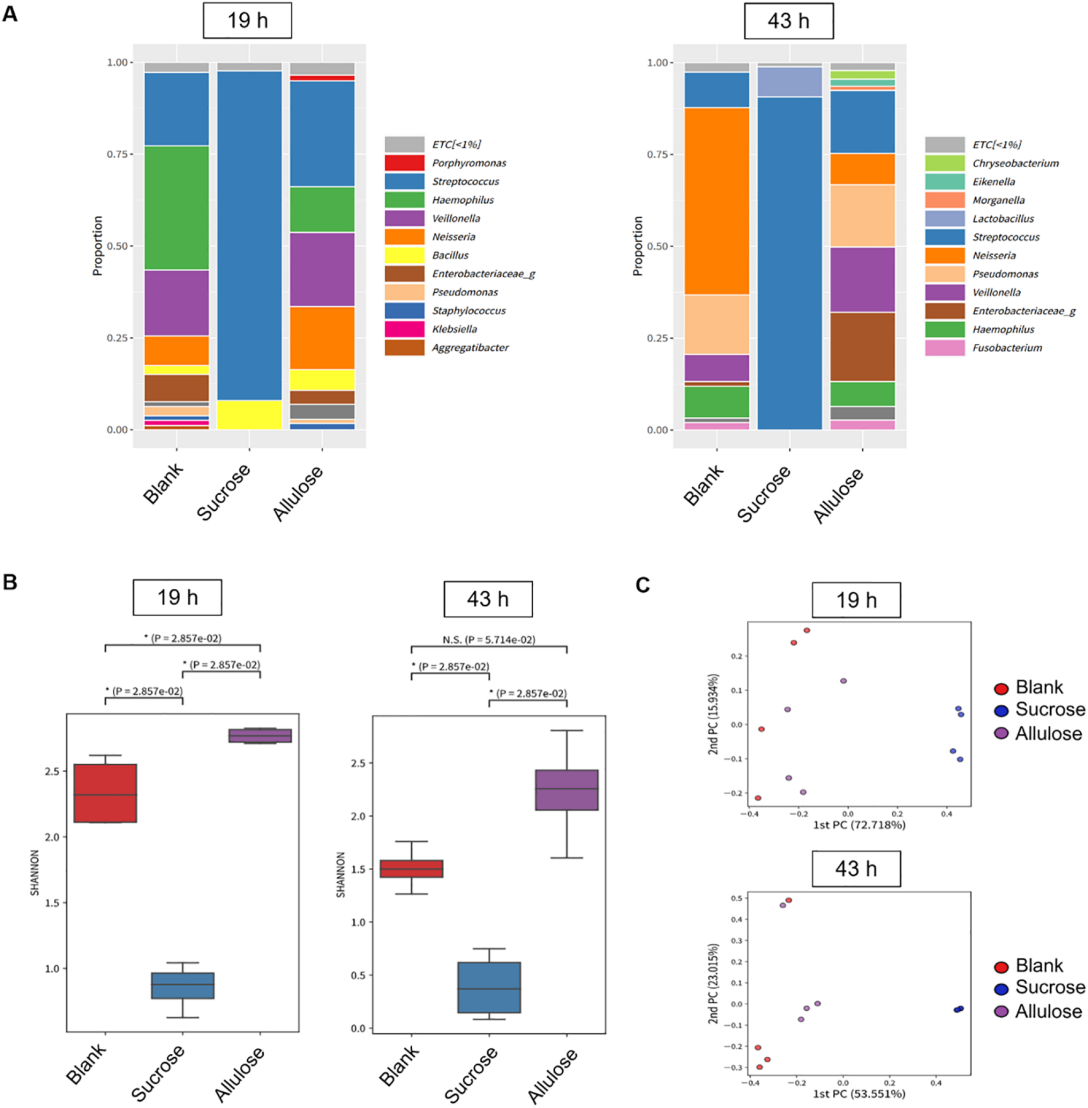
Microbial diversity and community structure in biofilm grown in the presence of 1% (w/v) sucrose or allulose. **(A)** Relative abundance of bacterial genera in 19 (left) and 43 h (right) biofilms grown on saliva-coated hydroxyapatite (sHA) discs with 1% (w/v) sucrose, allulose, or no sugar (blank). **(B)** Alpha-diversity (Shannon index) of microbial communities among treatment groups at 19 and 43 h. **(C)** Principal coordinate analysis (PCoA) of beta-diversity (Bray–Curtis distance) across treatment groups.

Following confocal imaging, diversity analysis showed that the bacterial community structure was distinct among the groups (**Figure 7**). Alpha-diversity, in terms of the Shannon index, which is a measure of both richness and evenness (Kitikidou et al., 2024), was significantly lower in the sucrose-supplemented biofilm than in the no-sugar-added control and allulose-supplemented biofilms in early and mature stages, respectively (**Figures 7A, B**; *p*=2.8E−2). Interestingly, allulose maintained the highest Shannon diversity index compared to the other two sweeteners at both 19 and 43 h. This suggests the ecological neutrality of allulose under the tested conditions by maintaining or promoting microbial diversity. Beta-diversity analysis (principal coordinate analysis based on the Bray–Curtis distance) revealed distinct clustering of microbial communities by treatment and time point. At both early (19 h) and mature (43 h) stages, sucrose-treated biofilms showed strong separation from both the allulose-supplemented and no-sugar-added control biofilms, indicating a marked shift in microbial community composition toward a dysbiotic state. In contrast, allulose-treated communities clustered closer to the no-sugar-added control communities, suggesting the maintenance of a more health-compatible microbial community (**Figure 7C**). Sucrose significantly reduced community diversity by selectively promoting the growth of acidogenic and aciduric *Streptococcus* and *Lactobacillus* species. In contrast, the relative abundance was maintained or increased under allulose supplementation compared to that without any supplementation (**Figure 7A**).

Biofilm development is a multistep process that begins with the attachment of early colonizers via specific or non-specific interactions with pellicle-coated surfaces (Marsh and Bradshaw, 1995; Dang and Lovell, 2000; Rickard et al., 2003). Favorable conditions promote microcolony formation by primary colonizers, subsequently facilitating secondary (late) colonizers to adhere primarily through co-aggregation (Rickard et al., 2003). Genera such as *Streptococcus*, *Actinomyces*, *Haemophilus*, *Veillonella*, and *Neisseria* are well-established early colonizers (Ritz, 1967; Foster and Kolenbrander, 2004; Dige et al., 2009; Huang et al., 2011). In this study, the presence of all these genera (except *Actinomyces)* in the allulose-treated and blank (saliva-origin microbiome) groups supported the establishment of a health-associated early biofilm community. *Veillonella* species convert the lactic acid produced by fermentative bacteria, such as *Streptococcus* sp., into weak acids, which may reduce the enamel demineralization rate (Geddes, 1972; Mikx and Van der Hoeven, 1975). The enrichment and maintenance of *Veillonella* sp. in the allulose-treated group than in the no-sugar-added group confirmed a more balanced community with metabolic cooperation (**Figure 7A**).

Additionally, the presence of *Neisseria*, *Haemophilu*s, *Granulicatella*, *Fusobacterium*, and *Veillonella* further supports the establishment of a healthy nitrate-reducing biofilm with a beneficial role in maintaining oral homeostasis (Hyde et al., 2014). Notably, *Fusobacterium* plays a key bridging role in plaque development, promoting co-aggregation and supporting anaerobic colonizers (Bradshaw et al., 1998; Kolenbrander et al., 1999; Rickard et al., 2003). Hence, its presence in the allulose-supplemented group suggests a diverse microbial community structure, where microbial connectivity and balance were preserved (**Figures 7A–C**).

Altogether, our microbial community analysis data align with the “ecological plaque hypothesis,” which explains that environmental shifts owing to acid production from dietary sugar fermentation can select acid-tolerating species such as *Streptococcus* and *Lactobacillus*, developing dysbiotic, caries-associated biofilms. By dealing with these detrimental and virulent factors, the allulose-treated microcosms retained microbial interactions, community diversity, and spatial organization, which are key characteristics of health-compatible biofilms.

In this study, we used a multi-tiered platform to investigate the ecological and functional effects of allulose on oral biofilm development. Using a biologically relevant model that included single-species planktonic, biofilm, dual-species biofilm, and saliva-derived microcosms, we systematically showed that allulose does not support the key virulence traits typically promoted by commonly used fermentable sugars such as sucrose, fructose, and glucose. Allulose consistently resulted in lower growth, acidogenicity, and EPS matrix formation compared to fermentable sugars, while preserving the ecological equilibrium of commensal bacteria (*S. oralis*). Additionally, it preserved many health-compatible taxa such as *Neisseria*, *Haemophilus*, *Granulicatella*, and *Veillonella* and maintained high alpha-diversity, indicating microbial equilibrium and ecological resilience. The follow-up experiment will apply shotgun metagenomics to identify species-level community shifts and functional gene analyses to link taxonomic shifts to cariogenic traits.

The overall findings of our study indicate that allulose is a microbiome-friendly sugar substitute that may limit cariogenic shifts under *in vitro* conditions. While promising, the translational potential of allulose as an alternative to established cariostatic agents, such as xylitol and erythritol, warrants further investigation.

We acknowledge the limitations of this platform, particularly the inability of the continuous sugar exposure model to accurately mimic the dynamic nature of dietary intake. Future research should aim to improve the ecological relevance of cariogenic evaluations by incorporating a feast–famine strategy. In addition, future research should evaluate the composition of plaques, the dynamics of salivary pH, and the incidence of caries over time to validate these *in vitro* results using *in vivo* models, including clinical trials. Ecological engineering of oral microbiota may also be clarified by further studies on the interactions of allulose with fluoride, oral prebiotics, or probiotics. Overall, this study establishes a strong foundation for developing next-generation microbiome-conscious sugar alternatives that support oral and systemic health.

## Data Availability

The datasets presented in this study can be found in online repositories. The names of the repository/repositories and accession number(s) can be found below: https://www.ncbi.nlm.nih.gov/genbank/, PRJNA1269248.
